# Increased Prognostic Yield by Combined Assessment of Non-Contrast Computed Tomography Markers of Antithrombotic-Related Spontaneous Intracerebral Hemorrhage Expansion

**DOI:** 10.3390/jcm11061596

**Published:** 2022-03-14

**Authors:** Aristeidis H. Katsanos, Himanshu Gupta, Andrea Morotti, Simon Beshara, Tushar Patil, Saeed Al-Zahrani, Georgios Tsivgoulis, Dariush Dowlatshahi, Joshua N. Goldstein, Andreas Charidimou, Ashkan Shoamanesh

**Affiliations:** 1 Department of Medicine (Neurology), McMaster University/Population Health Research Institute, Hamilton, ON L8L2X2, Canada; himanshu.gupta@medportal.ca (H.G.); saeed.alzahrani@medportal.ca (S.A.-Z.); ashkan.shoamanesh@phri.ca (A.S.); 2 Department of Neurological Sciences and Vision, Neurology Unit, ASST Spedali Civili, 25123 Brescia, Italy; andrea.morotti85@gmail.com; 3 Department of Medicine (Neurology), Queen’s Neurology, Kingston, ON K7L 3N6, Canada; simon.beshara@gmail.com; 4 Department of Neurology, Jawaharlal Nehru Medical College, Wardha 442005, India; tushar.patil@medportal.ca; 5 Second Department of Neurology, “Attikon” University Hospital, School of Medicine, National and Kapodistrian University of Athens, 124 62 Athens, Greece; tsivgoulisgiorg@yahoo.gr; 6 Department of Neurology, University of Tennessee Health Science Center, Memphis, TN 38163, USA; 7 Division of Neurology, Department of Medicine, University of Ottawa & Ottawa Hospital Research Institute, Ottawa, ON K1H 8M2, Canada; ddowlat@toh.ca; 8 Massachusetts General Hospital and Harvard Medical School, Boston, MA 02114, USA; jgoldstein@mgh.harvard.edu (J.N.G.); andreas.charidimou.09@ucl.ac.uk (A.C.)

**Keywords:** intracerebral hemorrhage, hematoma expansion, predictive score

## Abstract

Background and aims: The utility of proposed non-contrast computed tomography (NCCT) markers for the prediction of hematoma expansion in patients with antithrombotic-related spontaneous intracerebral hemorrhage (ICH) is limited. Additionally, there is significant overlap between different suggested ICH shape and density markers. Methods: We assessed the prognostic yield for hematoma expansion of a combined score incorporating features of ICH shape irregularity (satellite sign and/or Barras score ≥ 3), heterogeneous ICH density (swirl sign and/or Barras score ≥ 3) on baseline NCCT and timing from ICH onset to NCCT. Results: We evaluated data from 79 patients with antithrombotic-related spontaneous ICH (32% with hematoma expansion). Swirl (84% vs. 39%) and satellite signs (20% vs. 7%) on baseline NCCT were significantly more prevalent (*p* < 0.001) in patients with hematoma expansion. Patients with hematoma expansion had more irregular and heterogeneous bleeds on baseline NCCT scans, as quantified by higher (*p* < 0.001) Barras shape (4 (4–5) vs. 3 (2–4)) and density scores (4 (3–5) vs. 2 (1–3)), respectively. The overall diagnostic yield of the combined score (area under the curve: 0.86, 95%CI: 0.78–0.94) significantly outperformed (*p* < 0.001) the diagnostic yield of each individual marker. Scores of 4 or 5 in the combined score were associated with a sensitivity of 60.0%, specificity of 90.7%, overall diagnostic accuracy of 81.0%, positive likelihood ratio (LR) of 6.48, negative LR of 0.44, positive predictive value (PV) of 0.76 and negative PV of 0.83. Conclusion: Combined NCCT marker assessment seems to increase the prognostic accuracy for hematoma expansion in antithrombotic-related spontaneous ICH patients.

## 1. Introduction

Up to a third of patients with acute intracerebral hemorrhage (ICH) experience hematoma expansion during their hospitalization [1]. Hematoma expansion has been independently associated with both unfavorable outcomes and an increased risk of mortality [2]. The utility of imaging and clinical markers in the prediction of hematoma expansion is limited [3]. Specifically on non-contrast computed tomography (CT), there may be significant overlap between heterogeneous shape, density, and various non-contrast markers of ICH expansion [4]. Diagnostic accuracy of non-contrast CT markers has been variable in previous studies, with sensitivity and specificity values being suboptimal for any meaningful implementation in clinical practice or trial design [5].

In the present manuscript we evaluate the utility of a combined score, based on previously identified individual markers on non-contrast CT imaging, for the prediction of hematoma expansion during the first 24 h from hospital admission of patients presenting with acute antithrombotic-related spontaneous ICH.

## 2. Methods

We performed a post-hoc analysis of consecutive adult (≥18 years old) patients with spontaneous ICH and history of antithrombotic treatment (antiplatelet and/or anticoagulant) prior to the onset of the ictus admitted at a single tertiary care center (Hamilton General Hospital, Hamilton Health Sciences, Hamilton, ON, Canada) over a 6-year period from January 2010 to April 2016. The retrospective observational chart review of the patients with ICH admitted over this time period was approved by the Hamilton Integrated Research Ethics Board. Patients that had no repeated CT imaging available in the first 24 h from their admission were excluded. We also excluded cases that were determined to have secondary causes for intracranial bleeding or due to a final diagnosis of hemorrhagic transformation of an infarct. 

Patient records were reviewed for demographic information, vascular risk factors, antithrombotic treatment, and time from symptom onset to baseline CT acquisition. We reviewed the admission and follow-up non-contrast CT images of all eligible patients. ICH location on baseline non-contrast CT scans was coded as either lobar or deep (subcortical). We also screened for the presence of concomitant subdural, subarachnoid, or intraventricular hemorrhage. Intraventricular hemorrhage extension was scored with the use of the modified Graeb scale, as previously described [6]. The intraparenchymal hematoma shape and density was further characterized both as dichotomous (regular vs. irregular, homogenous vs. heterogeneous) and also with the corresponding Barras density and shape scores [7]. We also evaluated the presence of fluid level in the intraparenchymal hematoma, identified spots with lower attenuation inside the intraparenchymal hematoma (swirl sign) and/or satellite bleeding lesions adjacent to the primary intraparenchymal hematoma [4]. Images were rated by a single rater (AS) with substantial experience in the neuroimaging of ICH. Non-contrast CT imaging markers in ICH have previously been reported to have good-to-excellent inter-rater and intra-rater reliabilities, particularly for the evaluation of heterogeneity and hypodensities presence [8]. The hematoma volume of the intraparenchymal bleed was calculated at both baseline non-contrast CT on admission and repeat follow-up CT scan using a modified ABC/2 formula that accounts for the hemorrhage shape [9]. In cases with multiple repeated scans early during their admission, the closest scan to 24 h after baseline imaging was selected. Hematoma expansion was defined as an increase in ICH volume of ≥6 mL or ≥33% on repeat CT imaging compared to baseline non-contrast CT scan [2].

We compared all imaging characteristics between the patients with and without hematoma expansion within 24 h from hospital admission. Dichotomous and continuous variables were presented with the use of percentage and median values with corresponding interquartile ranges (IQRs). Differences between the two groups were assessed with the use of the chi-square test or Mann-Whitney U test, respectively. After comparing the non-contrast baseline CT characteristics between patients with and without hematoma expansion, we constructed a combined 5-point score based on data driven analysis summarizing the hematoma shape (1 point for irregular shape-Barras score III-V), hematoma density (1 point for heterogeneous density Barras score III-V, 1 point for presence of swirl sign), presence of satellite lesions (1 point for presence) and time from symptom onset to baseline CT acquisition (1 point if less than 6 h). Receiver operating characteristic (ROC) curve analysis was used to identify the optimal cut-off point of the constructed combined score for the prediction of hematoma expansion following hospital admission. The overall combined score predictive ability compared to the individual imaging markers was assessed by comparing the corresponding areas under the curve (AUCs) with the use of the Delong test. Sensitivity, specificity, overall diagnostic accuracy, positive likelihood (LR+), negative likelihood (LR−), positive predictive value (PPV) and negative predictive value (NPV) measures were derived from cross tabulations. All statistical analyses were conducted with the Stata Statistical Software Release 13 (College Station, TX, StataCorp LP, College Station, TX, USA).

## 3. Results

A total of 79 ICH patients (32% with hematoma expansion) met our eligibility criteria. Baseline clinical characteristics of included patients are provided in Table 1. Intracranial vascular imaging uncovered the presence of ruptured aneurysms in two patients and changes consisted of Moyamoya disease in another patient.

Patients with hematoma expansion had more irregular and heterogeneous bleeds on baseline non-contrast CT scan, as quantified by higher (*p* < 0.001) median Barras shape (4 (4–5) vs. 3 (2–4)) and density scores (4 (3–5) vs. 2 (1–3)), respectively. Both swirl (84% vs. 39%) and satellite signs (20% vs. 7%) on baseline non-contrast CT were significantly more prevalent (*p* < 0.001) in patients with hematoma expansion. Baseline hematoma volume was additionally associated with hematoma expansion (20.0 (IQR: 9.1–29.9) vs. 11.6 (IQR: 1.1–24.5), *p* = 0.044. Patients with hematoma expansion had a shorter duration of time between symptom onset and baseline CT (183 min (IQR: 92–523) vs. 560 min (223–1440), *p* = 0.005; Table 2). 

**Figure 1:**
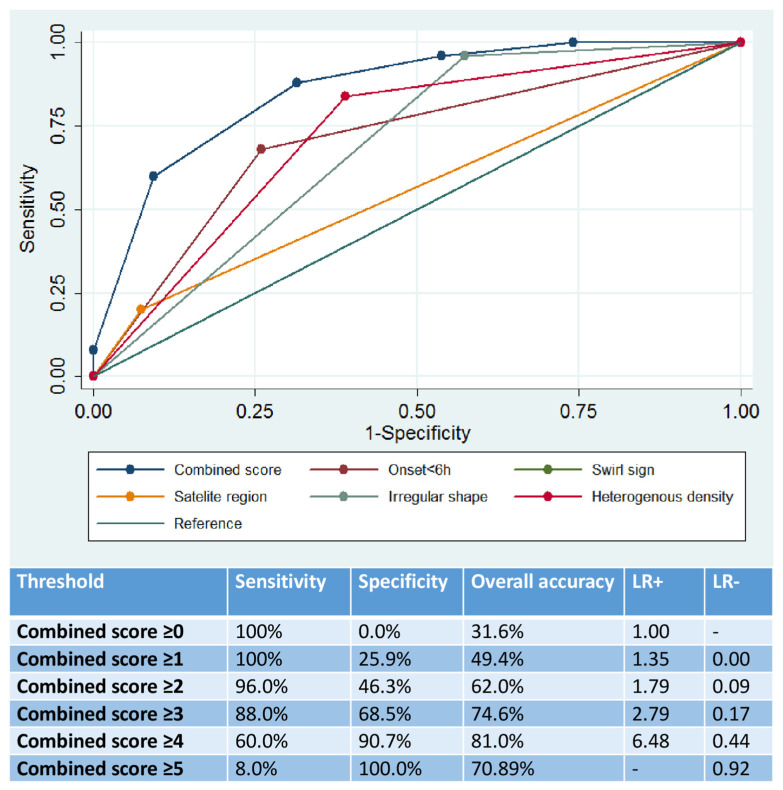
Prognostic utility of the combined score and individual non-contrast computed tomography imaging markers in the prediction of hematoma expansion.

The 5-point combined score had a good overall prognostic utility (AUC: 0.86, 95%CI: 0.78–0.94), which significantly outperformed the prognostic yield of each individual marker (*p* < 0.001 by DeLong test; Figure 1). Scores of 4 or 5 were associated with a sensitivity of 60.0%, specificity of 90.7%, overall diagnostic accuracy of 81.0%, LR+ of 6.48, LR− of 0.44, PPV of 0.76 and NPV of 0.83 (Figure 1).

## 4. Discussion

Our study showed that combined assessment of imaging markers in baseline non-contrast CT can increase the prognostic accuracy for hematoma expansion in ICH patients, when compared to the predictive ability of each individual marker. The overall predictive accuracy of our proposed score is comparable to the previously reported scores, with most of them providing high specificity but moderate sensitivity in the prediction of hematoma expansion [10]. However, our score is the first to combine markers of hematoma shape and density, assessed with the Barras scoring system, with time from symptom onset to CT imaging acquisition. Our model has a good accuracy in ‘ruling in’ hematoma expansion and indicates that patients with acute ICH who present within 6 h from symptom onset and are found to have irregular hematoma shape and heterogeneous density on their baseline CT scan (combined score of 4 or more), hematoma expansion is very likely. This information could potentially be useful in clinical decision-making, such as individualizing the intensity of clinical monitoring and most appropriate in hospital setting, as well as precise decision-making surrounding promising but yet to be established acute ICH treatments aiming to mitigate hematoma expansion (i.e., intensive systolic blood pressure lowering below 140 mm Hg or tranexamic acid). Additionally, the lack of requisite intravenous contrast administration make it a much more practical selection tool for identifying acute ICH patients at heightened risk of hematoma expansion for inclusion in future global multicenter treatment trials. 

We noted that patients with hematoma expansion had more irregular and heterogeneous bleeds. This is likely a manifestation of turbulence from ongoing intrahematomal bleeding and could represent secondary bleeding from shear injury to surrounding arterioles from a rapidly expanding hematoma (‘avalanche theory’) in the case of satellites. The primacy of shorter times from symptom onset to hospital presentation for the risk of hematoma expansion has previously been well described [11]. 

Notwithstanding the impact of hematoma expansion on worse ICH-related outcomes, it also constitutes an important clinical target for medical intervention [12]. The ability to identify patients at risk for hematoma expansion based on non-contrast CT features is not only of great clinical importance, to guide more careful monitoring and inform prognosis, but can also be used to refine the selection of participants that are at high risk for hematoma expansion in future therapeutic randomized trials [13]. Large phase III acute ICH randomized trials have mainly used early timing from symptom onset to randomization in their eligibility criteria to date in order to target ICH patients at heightened risk for hematoma expansion [14,15,16,17]. This approach has been limited by the lack of hematoma expansion in large proportions of trial populations, limiting the likelihood of success in demonstrating a favorable treatment effect and the exclusion of otherwise eligible candidates at substantial risk for hematoma expansion presenting in delayed timeframes or with unknown symptom onset times [18]. Using a pragmatic non-contrast CT based prognostic score utilizing both imaging markers and time from symptoms could lead to improved patient selection and maximize the likelihood of success in future acute ICH blood pressure lowering or hemostatic treatment trials. Some limitations of our work need to be acknowledged. First, it should be highlighted that this is a post-hoc analysis of a retrospective cohort study from a single institution. Moreover, we included patients presenting with spontaneous ICH and history of previous use of antithrombotics (antiplatelets and/or anticoagulants). Therefore, we cannot confirm with certainty whether our findings and the utility of the proposed combined score are applicable to other populations and clinical care settings. A recent systematic review highlighted that the real-world performance of hematoma expansion predictive scores in validation studies was significantly lower than their performance in derivation studies [3]. Additionally, given the variability in the assessment of imaging features of acute ICH between different evaluators, the interrater variability or our proposed score needs to be evaluated. Secondly, it should be noted that in the combined score, only imaging markers and timing from symptom onset to the acquisition of baseline non-contrast CT scan were included. Due to the limited sample size, the association of clinical factors, including antithrombotic treatment or blood pressure control, with hematoma expansion has not been evaluated in our cohort and by study design these factors were not accounted in the construction process of the combined imaging score. We should also acknowledge that 24-h follow-up scans are not routinely performed in every patient with acute ICH admitted to our institution. This could have introduced bias due to the exclusion of patients who deteriorated early or who became palliative soon after their admission. The presence of swirl sign, heterogeneous density and irregular hematoma shape have also been associated with poor clinical outcomes after adjustment for hematoma expansion [19]. Due to the lack of long-term functional outcome data in our cohort, we were not able to evaluate further the association of our combined score with patient clinical outcomes.

Our study suggests that a combined score including non-contrast CT markers and time from symptom onset can provide good accuracy for hematoma expansion. If externally validated, such a model could provide a valuable tool for participant selection in acute ICH treatment trials and individualized treatment decisions in patients with acute antithrombotic-related spontaneous ICH. 

## Figures and Tables

**Table 1:** Clinical characteristics of patients with acute intraparenchymal hemorrhage according to the occurrence of hematoma expansion.

Variable	Hematoma Expansion (*n* = 25)	No Hematoma Expansion (*n* = 54)	*p*-Value
Age, years, median (IQR)	76 (71–81)	74 (68–80)	0.434
Females (%)	24%	32%	0.465
Hypertension (%)	71%	67%	0.744
Diabetes mellitus (%)	75%	68%	0.579
Dyslipidemia (%)	81%	47%	0.016
Coronary artery disease (%)	50%	24%	0.041
Prior stroke (%)	44%	14%	0.008
Congestive heart failure (%)	19%	14%	0.622
Atrial fibrillation (%)	56%	34%	0.103
Current smoker (%)	7%	12%	0.586
Alcohol use (%)	14%	7%	0.381
Dementia (%)	19%	9%	0.238
Antiplatelet use (%)	67%	75%	0.422
Anticoagulant use (%)	48%	33%	0.212
Admission NIHSS, median (IQR)	10 (3–17)	4 (1–6)	0.102
Admission GCS, median (IQR)	15 (12–15)	15 (13–15)	0.359
Systolic BP on admission, mmHg median (IQR)	173 (148–190)	153 (133–189)	0.166
Diastolic BP on admission, mmHg median (IQR)	92 (89–101)	87 (75–95)	0.022
Time from onset to CT (min, median, IQR)	183 (92–523)	560 (223–1440)	0.005
Time from onset to CT< 6 h (%)	54.8	16.7	<0.001

IQR: interquartile range, NIHSS: National Institutes of Health Stroke Scale, GCS: Glasgow Coma Scale, BP: blood pressure, CT: computed tomography.

**Table 2:** Imaging characteristics of baseline non-contrast computed tomography scan of patients with acute intraparenchymal hemorrhage according to the occurrence of hematoma expansion.

Variable	Hematoma Expansion (*n* = 25)	No Hematoma Expansion (*n* = 54)	*p*-Value
Lobar hemorrhage (%)	40.0%	38.9%	0.925
SDH present (%)	20.0%	20.3%	0.967
IVH present (%)	48.0%	38.9%	0.445
IVH Graeb score (median, IQR)	2 (0–5)	2 (0–5)	0.726
SAH present (%)	40.0%	25.9%	0.206
Fluid level present (%)	16.0%	9.2%	0.380
Swirl sign present (%)	84.0%	38.9%	<0.001
Irregular hematoma present (%)	100%	79.6%	0.015
Barras shape score (median, IQR)	4 (4–5)	3 (2–4)	<0.001
Heterogeneous density present (%)	88.0%	59.2%	0.011
Barras density score (median, IQR)	4 (3–5)	2 (1–3)	<0.001
Combined Barras score (median, IQR)	7 (7–10)	5 (3–8)	<0.001
Satellite region of ICH (%)	20.0%	7.4%	0.101
ICH volume (cm^3^, median, IQR)	20.0 (9.1–29.9)	11.6 (1.1–24.5)	0.044

SDH: subdural hemorrhage, IVH: intraventricular hemorrhage, SAH: subarachnoid hemorrhage, IQR: interquartile range, CT: computed tomography.

## Data Availability

Data can be provided from the corresponding author upon reasonable request.
